# ‘It is a difficult topic’ – a qualitative study of midwives´ experiences with routine antenatal enquiry for intimate partner violence

**DOI:** 10.1186/s12884-017-1352-2

**Published:** 2017-06-02

**Authors:** L. Henriksen, L.M Garnweidner-Holme, K.K Thorsteinsen, M. Lukasse

**Affiliations:** 1 Faculty of Health Sciences, Department of Nursing and Health Promotion, Oslo and Akershus, University College of Applied Sciences, St. Olavs Plass, P.O. Box 4, 0310 Oslo, Norway; 2 Division of General Gynaecology and Obstetrics, Oslo University Hospital, P.O. Box 4950 Nydalen, N-0424 Oslo, Norway

**Keywords:** Intimate partner violence, Antenatal care, Routine enquiry, Midwifery care

## Abstract

**Background:**

Intimate partner violence (IPV) during pregnancy may jeopardize maternal and fetal health (IJFWM 49:159-164, 2004; IJGO 133:269-276, 2016). In recognition of the significant public health impact of IPV, the Norwegian Directorate of Health issued new guidelines in 2014, which recommend that health professionals routinely ask all women in antenatal care about their exposure to violence. The objective of this study was to gain an in-depth understanding of midwives’ experiences with routine enquiry for intimate partner violence during the antenatal period.

**Methods:**

The study had a qualitative design. Individual semi-structured interviews with eight midwives providing antenatal care at eight Mother and Child Health Centres (MCHC) in Norway were conducted. Graneheim and Lundmans method of content analysis inspired the analysis.

**Results:**

Three main themes emerged: *Midwives do ask about violence; It can be a challenge*; and *Factors that make it easier to ask.* All midwives enquired, but not on a regular basis, about violence. The midwives’ personal interest in the topic was an important factor that made it easier for them to ask about violence. Lack of time, fear of not knowing how to deal with a positive answer and lack of organizational support were barriers to asking pregnant women about their experiences of violence.

**Conclusion:**

Midwives were aware of the guidelines and made some efforts to implement them. However, further education and organisational support is needed to enable midwives to routinely ask all pregnant women about IVP.

**Electronic supplementary material:**

The online version of this article (doi:10.1186/s12884-017-1352-2) contains supplementary material, which is available to authorized users.

## Background

Intimate partner violence (IPV) during pregnancy is a severe public health problem that can jeopardize maternal and fetal health [1, 2]. Global prevalence estimates vary, usually due to differences in definitions, contexts, materials and methods used when examining violence [3–5]. In a recent meta-analysis of IPV during pregnancy of 92 studies from 23 countries, the average reported prevalence of emotional abuse was 28.4%, physical abuse was 13.8%, and sexual abuse was 8.0% [5]. In Norway, the prevalence varies from one to 5 % in different studies [3, 6–8]. The numbers are comparable with a new longitudinal cohort study done in Sweden [9]. Among 1573 women in this study, 2.5% reported violence during pregnancy [9]. The prevalence increased in the early postnatal period to 3.3% [9]. This finding is consistent with a longitudinal study in the UK showing an increase in IPV after pregnancy [10].

IPV prior to pregnancy, during pregnancy or in the new-born period is associated with adverse health outcomes like depression, miscarriage, stillbirth, preterm birth and low birth weight [2, 11, 12]. It may also affect the way women interact and connect with their babies [13].

In recognition of the significant public health impact of IPV, the Norwegian Directorate of Health recommended in new guidelines in 2014 in which health professionals should ask women attending antenatal care about exposure to violence with a few exceptions [14]. The most important of which is when the health personnel are not able to ask the woman in a private setting [14]. However, previous studies suggest that screening or routine enquiry about violence can contribute to a higher rate of violence disclosure and that antenatal care is a recommended setting [15]. This period is recognised as an ideal ‘window of opportunity’ to address IPV because pregnancy is a time when women are in regular contact with health care providers [16]. Pregnancy is an important context for safety planning, as child wellbeing and safety is a priority for many abused women [17]. For these reasons, women may be motivated for change [17].

Violence can be difficult to address for both the person who is exposed to it and for health professionals. Emotions like shame, judgment, uncertainty and lack of knowledge about violence can contribute to whether or not violence is discussed [18]. Studies have shown that barriers to asking questions about violence are the nature of the topic, lack of training and uncertainty about management after disclosure [19]; thus, health professionals themselves may be obstacles when it comes to addressing violence [20].

Women are not generally negative about being asked about violence [21]. A Cochrane review assessing the effect of IPV screening in healthcare settings reported no adverse outcomes for women because of the screening [15].

### Antenatal care in Norway

In Norway, almost every pregnant woman attends antenatal care, a free and well-integrated part of the public health system [22]. It is usually general physicians (GP) or midwives in primary healthcare that are responsible for antenatal care [22] and women are free to choose on or the other or both. The majority of midwives work at a Mother and Child Health Centre (MCHC). They are part of the public health care system and can be found in every community in Norway. In addition to midwives who provide antenatal care, public health nurses provide health services for the child. The overall aim of antenatal care is to ensure the wellbeing of the mother and fetus and identify complications [22]. The routine care is comprised of eight to ten visits and include screening for high blood pressure, various blood tests and the mapping of issues that can influence the pregnancy such as former pregnancy complications, previous and current health issues, smoking, consumption of alcohol, mental health and exposure to violence.

Some municipalities in Norway have incorporated questions about violence in antenatal care and participated in a project called ‘Early involvement’ (Norwegian: *Tidlig inn*) [23], which was initiated by the Norwegian Health Directorate [23]. The overall goal is to enhance competence on mental health issues, drug and/or alcohol abuse and intimate partner violence among professionals working with families and children and to make them more confident regarding early identification and intervention [23]. Midwives and GPs in primary care were among the target group and were invited to participate in special training. An evaluation after the project was implemented showed that only one-third of the attendees said that they asked about violence after the course. In contrast, two-thirds of the leaders said the program had been introduced and adapted [23]. This may suggest that aspects other than training affect whether or not healthcare providers ask about violence. Thus, the objective of this study was to gain an in-depth understanding of midwives’ experiences with routine enquiry for intimate partner violence during the antenatal period.

## Methods

### Data collection

A qualitative approach with individual semi-structured interviews was chosen for data collection because we wanted to gain deeper insight into personal experiences with the communication of a sensitive topic [24]. It was also preferred instead of, for example, a survey with qualitative responses because of the opportunity to elaborate on and examine answers during the interview. Eight midwives were purposively recruited from eight Mother and Child Health Centres (MCHC) in Oslo and a smaller town in another part of Norway. This approach was used to recruit midwives with different experiences asking about violence and to ensure diversity regarding ethnicity and socio-economic status between the MCHCs. Recruitment was carried out until the desired richness of individual cases was reached [24]. The study received ethical approval from the Norwegian Social Science Data Service (Nr 48,640). Midwives from different MCHCs were contacted by e-mail and invited to participate in the study. They were given written and oral information about the aim of the study, including detailed information about the topic, and they were assured that all data would be treated confidentially and that they could withdraw at any time. Contact information for the research group was provided in the written information if they needed to discuss something after the interviews. All midwives provided written informed consent. An interview guide with open-ended questions was prepared in advance to keep the conversation within the boundaries of the chosen subject. The main themes of the interview were knowledge regarding violence and the guidelines, talking about violence with the pregnant woman and organisational and workplace factors. The interview guide is provided as Additional file 1: Table S1.

One member of the research team (KKT) performed the interviews, which were all conducted at the MCHC where the midwives worked. The interviews lasted between 50 and 90 min.

### Data analysis

The interviews were recorded and transcribed verbatim by researcher KKT. We used content analysis, inspired by Graneheim and Lundman, to analyse the data [25]. The analysis was performed in four steps: 1) thoroughly reviewing of the interviews to obtain an idea of the content; 2) dividing the text into meaning units (sentences or paragraphs in the text that related to each other and to the aim of the study); 3) condensing the meaning units and labelling them with codes, which were then distributed into categories that were abstracted and compared for similarities and differences and condensed into subthemes; and 4) analysing the subthemes and unifying them into three main themes. To strengthen trustworthiness, the condensed meaning units, codes, subthemes and themes were discussed by authors KKT and LH throughout the analysis process until agreement was reached. Examples of analysis with meaning units, codes, subthemes and theme are given as Additional file 1: Table S2. Quotations were chosen to represent the range of views for each theme. We have followed the consolidated criteria for reporting qualitative research (COREQ) [26].

## Results

### Characteristics of the study participants

Eight midwives with between 3 and 30 years of work experience participated. The majority had both hospital (labour wards) and antenatal care experience. Five of the midwives worked in antenatal care only, and three had combined positions and worked both in antenatal care and in hospitals in labour wards. Table 1 summarizes midwives’ characteristics related to their working experience, training and communication about violence.

**Table 1:** Midwives characteristics

Midwife	Years of experience as a midwife	Workplace	IPV Training	Length of time enquiring about IPV
1	12	MCHC	Yes	1 year
2	30	MCHC/Hospital	Yes, partial	3 months
3	10	MCHC	Yes, extensive	3–4 years
4	12	MCHC/Hospital	No	3 months*
5	3	MCHC/Hospital	No	3 months*
6	23	MCHC	No	6 months*
7	30	MCHC	Yes, extensive	4 years
8	10	MCHC	Yes, partial	9 months

*Have not implemented routine enquiry, practice case findings

### The midwives’ experiences of routine enquiry about IPV in antenatal care

Two of the midwives had participated in ‘Early involvement’, which means that they were asking about violence exposure before the introduction of the new guidelines. However, they stated that they had done it more systematically for the last two years. The rest of the midwives had between three months and one year experience asking pregnant women about violence. Five said they had received some training in how to ask about IPV.

Data analysis revealed three main themes: 1) *Midwives do ask about violence*; 2) *It can be a challenge*; and 3) *Factors that make it easier to ask*. The themes and subthemes are presented in Table 2 below.

**Table 2:** Overview of themes and subthemes

Theme	Subtheme
Midwives do ask about violence	Motivation Knowledge and attitudes It was difficult to start asking
It can be a challenge	Sensitive topic Lack of resources The woman does not attend antenatal care alone Fear of revealing
Factors that make it easier to ask	Alternative approaches Engagement Sensitive communication Positive feedback from women

### Midwives do ask about violence

All midwives in this study reported that they ask about violence, but they do not routinely ask all women. They reported that the guidelines’ recommendation to ask everybody was challenging and following the recommendation depended upon the situation. We identified three subthemes within this theme: *motivation, knowledge and attitudes, and it was difficult to start asking.*

*Motivation:* The midwives´ motivations to ask about violence differed. The majority were motivated because they believed in asking as an instrument to identify violence and potentially support women and keep them safe. Midwife 1 said:

*(...), but the value in asking, the relevance and the aspect of preventing harm, it is so important and huge, it becomes worth it anyway.*

Some midwives felt provoked by the new guidelines because they were imposed upon to perform a new task for which they did not feel equipped. They were motivated because they felt it was their moral duty, not because they agreed with the guidelines. They mainly disagreed with the guidelines regarding the fact that they should ask every woman in antenatal care with few exceptions.

*Knowledge and attitudes:* Knowledge about and attitudes towards violence differed among the midwives. Some believed they worked in an area with less violence because the women they cared for were highly educated women and presented an overall orderly facade. They did not consider them to be in a high-risk group. Because of this, they did not think it was necessary to ask everybody. Some also said they did not believe that the women would disclose violence when asked. Midwife 4 said:

*Well, you can find violence in all parts of society, but I do not feel that our women are among the most deprived people. Thus, it’s not … These are not people who have a lot of issues, neither economic nor other problems.*

*It was difficult to start asking:* Difficulty starting a conversation about violence was commonly expressed by the midwives, which implies that it was not easy in the beginning, both on a personal and an organisational level. Two of the midwives clearly expressed frustration about the lack of consistency between the importance of the guidelines and the policy they experienced at their workplace. They were frustrated with the increasing workload without proper training, and they did not feel they had the time or the skills to deal with this new task. As Midwife 2 said:

*I feel that this is something we just have to deal with without anyone telling us how to do it. So I think that I feel provoked that they have just decided this without training us properly.*

### It can be a challenge

In the theme *It can be a challenge,* midwives highlighted that violence is a difficult topic regardless of the experience in asking, motivation and training they had received. The sensitive nature of the topic made it challenging to ask every woman. They emphasized the need for organisational structures and a support system, and they felt this was lacking before the guidelines were introduced. Four subthemes emerged within this theme: *sensitive topic*, *lack of resources*, *the woman does not attend antenatal care alone*, and *fear of disclosure.*

*Sensitive topic:* The midwives felt that the topic was sensitive, even those who said they had several years of experience talking about violence. Midwife 8 reflected upon how she felt it when she asked about violence:

*You don’t feel confident talking about it, but you do it anyway. Maybe you never will because it is difficult and sensitive.*

Others mentioned that the topic is still taboo and several talked about an underlying respect in society that the home is private, and what happens at home is a family business. This made it challenging to ask. Midwife 1 said:

*The subject is taboo. It’s a topic that has not been talked about, it has been treated with silence, and we respect what happens within the private home. I think that is just something we have learned from childhood.*

*Lack of resources:* All of the midwives talked about lack of time as the biggest challenge when it came to asking about violence. This was independent of whether or not they said they asked all women. Midwife 5 said:

*I have more than enough to do without digging too deep.*

Everyone was aware that violence was something that had to be addressed if uncovered, and that they would have to make time to deal with it if it came up. The uncertainty about what they could uncover and how it would affect other tasks was an issue among several of the midwives. Some of them also said that they could forget to ask about violence if they were busy. Midwife 4 said:

*The topic is big and difficult. It is big and difficult and takes time, right? And you know, if somebody discloses things you need to make time to address it.*

*The woman does not attend antenatal care alone:* The majority of the midwives said it was difficult to address violence as a topic if the woman came with her partner, a friend or a relative. In that case, they usually omitted the questions, and some of them said they probably did not ask the woman again later. The most experienced midwives said they talked about violence if the women came with her partner, but as a more general topic. Midwife 6 said:

*I don’t see that there is any reason to ask again. That would feel very awkward.*

*Fear of disclosure:* Several of the midwives expressed a fear about asking women about violence because they were afraid of the responsibility they have to assume if the woman disclosed violence. They were not certain if they could provide the right support and help the woman needed. Some lacked guidelines and a clear procedure at their workplace for dealing with disclosed violence. As Midwife 2 said:

*You are afraid of the answer. Because if you are inexperienced and have no training … I feel that I don’t have enough knowledge. How can I ensure that the woman gets the help she needs? I can refer her to other people, but I still have to support her immediately. I am not competent.*

Some of the midwives had uncovered serious violence in the past and become an important support person for the woman. They expressed a feeling of responsibility and fear for both the woman’s and their own safety.

### Factors that make it easier to ask

The last emerging theme was *Factors that make it easier to ask*. The midwives mentioned different tools that helped them to talk about violence, and four subthemes were identified: *alternative approaches, engagement, sensitive communication,* and *positive feedback from women.*

*Alternative approaches:* The midwives described different approaches when they prepared to ask about violence. Some started with other topics like depression or alcohol use before they asked about violence, or they had printed agendas with an overview of the topics that antenatal care covered that included violence. Another approach was an attempt to make the topic more harmless by telling the women that this is a routine enquiry and everybody is asked the same questions. The more experienced midwives said they did not need a predefined strategy to approach the topic; they were confident talking about violence. Midwife 1 said:

*I just ask as if this the most natural thing and I pretend to be very experienced in asking.*

*Engagement:* Those who expressed an interest in the topic were more confident about communicating about violence regardless of how long they had been asking. The midwives who were most experienced in asking had started to ask about violence before the guidelines were introduced and expressed long-lasting personal engagement. Some of the inexperienced midwives also described a personal engagement that helped them to ask questions despite insecurity and lack of proper training. Midwife 8 said:

*Then, personally, I became very engaged or interested in it; we must simply start to ask. It is so important to detect violence. We need to show women that we can handle the answers.*

*Sensitive communication:* All of the midwives agreed that it was important to be sensitive when talking about violence, and they used all of their midwifery skills when they addressed the topic. They expressed that this helped them build up a trustful relationship with the women, which was something they thought should be present when asking about violence. Because of this, their approaches differed when they made routine enquiries; some asked at the first consultation, and some said they waited to be able to gain trust. The midwives also talked about the importance of being sensitive in the sense of reading a woman’s body language, listening to the unsaid and acknowledging that things could be different from the way they appeared. Midwife 5 said:

*I feel that I am listening to a lot more than what they say; I listen to the whole body, expressions and the unsaid. And then I always think that, here, there are things here that do not come forward.*

*Positive feedback from women:* In the midwives’ experience, women do not seem to mind being asked about violence. They said that the majority answered questions regarding violence like every other question. When they saw that a woman opened up, it was easier for them to continue to ask questions. Some of the midwives were surprised that women expressed gratitude because somebody had asked about violence. Midwife 8 said:

*And that was what I discovered, when we dare to ask, when we dare to open up and perhaps demonstrate that we can handle this, the answers, then they say yes. Much more often than what I would have thought.*

## Discussion

The aim in this study was to explore midwives’ experiences with routine enquiry about IPV in the antenatal care context. Analysis of the semi-structured interviews revealed three main themes: *Midwives do ask about violence; It can be a challenge;* and *Factors that make it easier.*

*Midwives do ask about violence:* All of the midwives in this study had started to ask women about violence as a part of routine antenatal care, and the majority found it expedient to ask. However, some of the midwives had not implemented routine enquiry about violence. The Norwegian guidelines, in agreement with international guidelines [27], emphasise the importance of asking all women about violence with few exceptions [14]. According to the Norwegian Directorate of Health, the systems’ level of care, the political and organizational support, the clinical guidelines and training necessary to introduce routine enquiring to be ethical and safe [28] exists in Norway [14]. Since the responsibility for both the training and development of the clinical guidelines lays on each municipality [14], the introduction of the guidelines among the MCHCs has been random. This may be why the midwives in this study expressed both personal and organisational challenges that affected whether or not they asked all women. Hence, the guidelines from 2014 were not fully implemented in their antenatal care routines. When this study was conducted, more than two years had passed since the special guidelines concerning violence were introduced [14]. Violence and sexual abuse are a topic in the existing guidelines that covers all aspects of antenatal care in Norway (released in 2005) [22]; the importance of focusing on violence is not new. In a recent survey among 398 midwives in Norway, 41% said they ask all women about violence, approximately 30% asked half of the women and 11% did not ask at all [29]. Thus, our findings reflect the results of a large-scale quantitative survey.

One of the sub-themes within *Midwives do ask about violence* was knowledge and attitudes, and we saw that some of the reasons for not asking about violence were that the midwife did not suspect violence and did not think it occurred among their well-educated patients. This can indicate a misconception about violence that may be due to lack of training. Several studies indicate that health professionals lack knowledge about violence and its risk factors, and because of this, they underestimate the prevalence of violence [19, 30]. This is supported in the survey among the Norwegian midwives where the majority reported ‘no suspicion’ as the major reason for not asking pregnant women about violence [29]. The fact that there has been no standardized system for the implementation of the guidelines may be a reason why the midwives views regarding violence and whether all women should be asked on a routine basis differ. Other studies support the lack of standardized training as a reason why health professionals do not ask about violence (20, 21). That and a defined implementation period may also explain why several of the midwives in our study thought it was difficult to start asking about violence and why some did not have the motivation or skills to ask about violence.

*It can be a challenge:* The midwives in this study expressed challenges and barriers that made them less confident about asking about violence. The majority expressed an insecurity regarding what to do if a woman revealed that she lived in an abusive environment. This is not an unknown issue and Eustase et al. call this the ‘big fear factor’ in a study among Australian midwives [31]. This fear can prevent midwives from addressing violence. The Norwegian guidelines emphasize that the different municipalities and MCHCs need to have a clear protocol for the healthcare professionals to follow if violence is revealed [14]. The majority of the midwives in this study sought protocols like this. Since this was lacking, they did not feel supported at their workplace and this added to their anxiety around potential disclosures.

Lack of time during consultations was a barrier for the midwives when it came to talking about IPV. If they uncovered violence, they were all aware that addressing the issue properly would require time they did not necessarily have, so they simply do not ask. Midwives in other studies also expressed worries regarding time [20, 31–33]. In the recent national survey of Norwegian midwives, time issues were a major reason why they did not ask all women about violence [29].

The majority of midwives did not ask about exposure to violence if the partner or a relative accompanied the women for the consultation. This is also expressed in other studies [23, 30, 33] and is in line with the guidelines that recommend the routine enquiry to be performed when the woman comes in alone [14]. The guidelines suggest that the midwife should encourage the woman to come alone to at least one check-up so that she can ask about IPV [14]. This conflicts with the general encouragement towards the partner being part of the pregnancy and attending antenatal care appointments [34, 35], and it may be a dilemma for the midwives. This was also a finding in a study among Swedish midwives [36]. However, some of the midwives in our study talked about violence despite the partner being present, but adjusted the questions and talked about the topic in more general terms. Regardless, the safety of the women must be the priority and this is why direct questioning about violence exposure should be performed when the woman is alone [14, 27.

*Factors that make it easier to ask:* To create a relationship with the women before addressing the violence issue was a factor that made it easier to ask about violence. This is supported by findings from several other studies [20, 23, 31, 37]. A trustful relationship with the midwife may help women reveal something as difficult as violence; pregnant women themselves also found this to be important [8]. As a consequence, the need to establish a relationship could make midwives wait before asking; LoGiudice found this to be true in a meta-synthesis in 2015 [33]. She pointed out that the need to build trust could sometimes be at the expense of asking early enough and that important time could pass by the time midwives had gained trust [33]. The Norwegian guidelines [14] and international guidelines [27] recommend incorporating questions about violence as early as possible in the pregnancy and repeating them in both the second and third trimesters. This can be difficult to follow in a Norwegian setting as midwives often work part time in antenatal and midwifery resources are scares in several municipalities [34]. The lack of midwifery resources may lead to women meeting midwives late in the pregnancy; hence, they do not have time to build up the trust it takes to ask about violence. A Norwegian survey supported this as it showed that midwives who worked part time in antenatal care (less than 25%) asked fewer women about violence, and they uncovered less violence [29].

Some midwives expressed that personal engagement seemed to help them when they implemented the new guidelines. Because they were committed, they expressed that it was easier both to enquire and to talk about violence. This was regardless how much training they got before they began following the guidelines. Others support the importance of a personal commitment by the midwife when addressing violence [20, 31]. In a qualitative study among different healthcare personnel in Norway, regardless of where they worked or how they addressed violence, Danielsen et al. found that personal commitment to the topic was important [23]. If a personal engagement is of great importance, it might explain why increased training is not necessarily enough to get midwives to routinely ask about violence. In a Norwegian project where they introduced routine enquiry about violence in a small part of Norway in 2007, approximately 50 present of the pregnant women were asked [8]. This was in spite of a thorough implementation, training and access to guidance throughout the period [8]. Taft et al. found similar screening numbers among maternal and child health nurses in Australia [37].

The majority of the midwives in the 2007 project were positive about asking about violence after the project period even though they only asked 50% of the pregnant women during the project period [8]. This suggests that time is important when implementing new guidelines and that attitudes can change. That time and experience can change the attitudes towards violence is supported by Baird et al. [38], who found a significant change of attitude in relation to how midwives saw their own role in uncovering violence in a follow-up study of a mandatory training program for midwives in England [38]. Five years after the training, all midwives considered asking about violence an integral part of their work, versus when the program started [38].

The participants experienced being a midwife as a tool to ask about violence because in this role they had a sensitivity and proximity towards the women they cared for. They said that they used all their midwifery skills when talking to women, and this helped them be sensitive when they communicated about difficult topics. Mauri et al. support this in a qualitative study where the participating midwives highlighted the importance of being sensitive, listening and paying attention to the women when detecting violence [39]. Midwives in Norway are used to asking about sensitive topics like smoking, body weight, alcohol and mental health [22], and the participants in our study expressed that they sometimes used those topics as a tool to make it easier to ask about violence. This may suggest that questions about violence are still new and given more time and experience, the midwives can be confident in talking about violence as a natural part of antenatal care.

### Limitations and methodological considerations

In this study, a qualitative design was chosen to gain a deeper understanding of a phenomenon; hence, this study was conducted with only a few participants. The results are derived from the participants’ reported experiences and may be transferrable to similar groups but cannot be generalized [40]. Throughout the process, measures to ensure trustworthiness, essential for others to judge the value of the study [40], have been taken. These include the use of an established and clearly described method for data analysis [25] and the use of direct quotations from the participants to help readers judge reliability for themselves [40]. More than one researcher has read the data and participated in the analysis; consensus regarding content and themes was achieved [40].

## Conclusion

This study provides insight into how Norwegian midwives experience routine enquiry into intimate partner violence in the antenatal care context. The midwives in this study did ask pregnant women about violence exposure, but not necessarily all women or on a routine basis. Thus, even though the guidelines regarding routine enquiry have existed for over two years, they are not fully implemented. Findings in this study indicate that midwives’ personal engagement regarding violence is important and creating a supportive environment to facilitate engagement and knowledge about how to ask may make it easier for midwives. They emphasized the need for education and organizational structures and support systems at their workplaces. Protocols and referral to existing services should be in place at every antenatal care clinic. Discussing communication strategies and plans of action and sharing experiences on a regular basis may provide midwives with more confidence regarding asking about violence. These are all measures that can help sustain routine enquiry for violence exposure as a clinical practice.

## Additional file

Additional file 1: Table S1. Interview guide; Description of data: English language copy of the interview guide used to direct the semi-structured interviews in this study. **Table S2.** Analysis process: Example of the with meaning units, codes, subthemes and theme. (DOCX 14 kb)

## Abbreviations

GP: General physicians
IPV: Intimate partner violence
MCHC: Mother and Child Health Centres

## References

[1] Boy A, Salihu HM (2004). Intimate partner violence and birth outcomes: a systematic review. Int J Fertil Womens med.

[2] Hill A, Pallitto C, McCleary-Sills J, Garcia-Moreno C (2016). A systematic review and meta-analysis of intimate partner violence during pregnancy and selected birth outcomes. Int J Gynaecol Obstet.

[3] Sorbo MF, Grimstad H, Bjorngaard JH, Schei B, Lukasse M (2013). Prevalence of sexual, physical and emotional abuse in the Norwegian mother and child cohort study. BMC Public Health.

[4] Finnbogadottir H, Dykes AK, Wann-Hansson C (2016). Prevalence and incidence of domestic violence during pregnancy and associated risk factors: a longitudinal cohort study in the south of Sweden. BMC Pregnancy Childbirth.

[5] James L, Brody D, Hamilton Z (2013). Risk factors for domestic violence during pregnancy: a meta-analytic review. Violence Vict.

[6] Lukasse M, Henriksen L, Vangen S, Schei B (2012). Sexual violence and pregnancy-related physical symptoms. BMC Pregnancy Childbirth.

[7] Lukasse M, Schei B, Ryding EL, Bidens Study G (2014). Prevalence and associated factors of fear of childbirth in six European countries. Sex Reprod Healthc.

[8] Hjemdal OK, Engnes K (2009). Å spørre om vold ved svangerskapskontroll. [Asking about violence in antanatal care (in Norwegian)].

[9] Finnbogadottir H, Dykes AK (2016). Increasing prevalence and incidence of domestic violence during the pregnancy and one and a half year postpartum, as well as risk factors: a longitudinal cohort study in southern Sweden. BMC Pregnancy Childbirth.

[10] Bowen E (2005). Heron J, Waylen a, Wolke D and the Alspac study team. Domestic violence risk during and after pregnancy: findings from a British longitudinal study. BJOG Int J Obstet Gynaecol.

[11] Taillieu TL, Brownridge DA, Tyler KA, Chan KL, Tiwari A, Santos SC (2016). Pregnancy and intimate partner violence in Canada: a comparison of victims who were and were not abused during pregnancy. J Fam Violence.

[12] Alhusen JL, Ray E, Sharps P, Bullock L (2015). Intimate partner violence during pregnancy: maternal and neonatal outcomes. J Women's Health.

[13] Hooker L, Samaraweera NY, Agius PA, Taft A (2016). Intimate partner violence and the experience of early motherhood: a cross-sectional analysis of factors associated with a poor experience of motherhood. Midwifery.

[14] Norwegian Directorate of Health: Nasjonal faglig retningslinje for svangerskapsomsorgen - hvordan avdekke vold. [National guidlines, ananatal care – how to uncover violence (in Norwegian)]. Oslo: The Norwegian Directorate of Health; 2014.

[15] O'Doherty L, Hegarty K, Ramsay J, Davidson LL, Feder G, Taft A (2015). Screening women for intimate partner violence in healthcare settings. Cochrane Database Syst rev.

[16] Devries KM, Kishor S, Johnson H, Stockl H, Bacchus LJ, Garcia-Moreno C, Watts C (2010). Intimate partner violence during pregnancy: analysis of prevalence data from 19 countries. Reprod Health Matters.

[17] Vatnar SKB, Bjørkly S (2010). Does it make any difference if she is a mother? An interactional perspective on intimate partner violence with a focus on motherhood and pregnancy. J Interpers Violence.

[18] Sprague S, Madden K, Simunovic N, Godin K, Pham NK, Bhandari M, Goslings JC (2012). Barriers to screening for intimate partner violence. Women Health.

[19] Feder G, Davies RA, Baird K, Dunne D, Eldridge S, Griffiths C, Gregory A, Howell A, Johnson M, Ramsay J (2011). Identification and referral to improve safety (IRIS) of women experiencing domestic violence with a primary care training and support programme: a cluster randomised controlled trial. Lancet.

[20] Finnbogadóttir H, Dykes A-K (2012). Midwives' awareness and experiences regarding domestic violence among pregnant women in southern Sweden. Midwifery.

[21] Hatcher AM, Woollett N, Pallitto CC, Mokoatle K, Stockl H, Garcia-Moreno C. Willing but not able: patient and provider receptiveness to addressing intimate partner violence in Johannesburg antenatal clinics. J Interpers Violence. 2016; [Epub ahead of print] 10.1177/0886260516651094 27215666

[22] Norwegian Directorate of Health: Nasjonal faglig retningslinje for svangerskapsomsorgen. [National guidlines, ananatal care. (in Norwegian)]. Oslo: The Norwegian Directorate of Health; 2014.

[23] Danielsen EM, Solberg A, Grøvdal Y (2016). Kommunal helse- og omsorgstjenesters arbeid med vold i nære relasjoner. En kvalitativ intervjuundersøkelse. [working with violence in primary care. Qualitative Interwievs. (in Norwegian) ].

[24] Green J, Thorogood N (2009). Qualitative methods for health research.

[25] Graneheim UH, Lundman B (2004). Qualitative content analysis in nursing research: concepts, procedures and measures to achieve trustworthiness. Nurse Educ Today.

[26] Tong A, Sainsbury P, Craig J (2007). Consolidated criteria for reporting qualitative research (COREQ): a 32-item checklist for interviews and focus groups. Int J Qual Health Care.

[27] WHO: Responding to intimate partner violence and sexual violence against women: WHO clinical and policy guidelines. In. Geneva: The Norwegian Directorate of Health; 2013. 24354041

[28] García-Moreno C, Hegarty K, d'Oliveira A. F. L, Koziol-MacLain J, Colombini M, Feder G. The health-systems response to violence against women. Lancet 2014;385, 1567-1579. 10.1016/S0140-6736(14)61837-7 25467583

[29] Henriksen L (2017). Spør norske jordmødre alle gravide om vold? [Does Norwegian midwives ask pregnant women about violence? (In Norwegian)]. Tidsskrift for jordmødre.

[30] Baird KM, Saito AS, Eustace J, Creedy DK (2015). An exploration of Australian midwives' knowledge of intimate partner violence against women during pregnancy. Women Birth.

[31] Eustace J, Baird K, Saito AS, Creedy DK (2015). Midwives’ experiences of routine enquiry for intimate partner violence in pregnancy. Women Birth.

[32] Hooker L, Small R, Taft A (2016). Understanding sustained domestic violence identification in maternal and child health nurse care: process evaluation from a 2-year follow-up of the MOVE trial. J adv Nurs.

[33] LoGiudice JA (2015). Prenatal screening for intimate partner violence: a qualitative meta-synthesis. Appl Nurs res.

[34] Norwegian Departement of Health: White paper nr 12 (2008–2009): En gledelig begivenhet – Om en sammenhengende svangerskaps-, fødsels- og barselomsorg. [A happy occation – continuity in maternity care. (In Norwegian)] Oslo: The Norwegian Directorate of Health; 2009.

[35] Ellberg L, Hogberg U (2010). Lindh V: 'We feel like one, they see us as two': new parents' discontent with postnatal care. Midwifery.

[36] Stenson K, Sidenvall B, Heimer G (2005). Midwives' experiences of routine antenatal questioning relating to men's violence against women. Midwifery.

[37] Taft A, Hooker L, Humphreys C, Hegarty K, Walter R, Adams C, Small R (2015). Maternal and child health nurse screening and care for mothers experiencing domestic violence (MOVE): a cluster randomised trial. BMC med.

[38] Baird K, Salmon D, White P (2013). A five year follow-up study of the Bristol pregnancy domestic violence programme to promote routine enquiry. Midwifery.

[39] Mauri EM, Nespoli A, Persico G, Zobbi VF (2015). Domestic violence during pregnancy: midwives experiences. Midwifery.

[40] Malterud K (2001). Qualitative research: standards, challenges, and guidelines. Lancet.

