# Supplementary material for: ‘It is a difficult topic’ – a qualitative study of midwives´ experiences with routine antenatal enquiry for intimate partner violence
Source: BMC Pregnancy Childbirth. 2017 Jun 2;17:165. doi: 10.1186/s12884-017-1352-2 (PMC5457554; doi:10.1186/s12884-017-1352-2)
Supplement: Additional file 1: Table S1. — Interview guide; Description of data: English language copy of the interview guide used to direct the semi-structured interviews in this study. Table S2. Analysis process: Example of the with meaning units, codes, subthemes and theme. (DOCX 14 kb) [file 12884_2017_1352_MOESM1_ESM.docx]

**Table S1: The interview guide**

| **Interview guide** |
| --- |
| **Can you tell me about your background as a midwife?**  How long have you worked as a midwife?  Where have you worked as a midwife?  What is your experience from antenatal care?  Can you tell about your experience regarding working with violence?  What do you associate with violence and pregnancy?  Are there aspects in antenatal care you find difficult to talk about? |
| **Can you tell me how you experience the routine enquiry about violence?**  The biggest challenges?  Why is it a challenge?  What is important to you as a midwife to start asking?  How to you gain trust?  Do you have special techniques that you use? If yes, can you describe them?  Dose it occur that you do not ask? Why?  Can you tell me about the last time you asked a women if she was expose to violence and got a positive answer?  Can you tell me about one time you think you handled asking in a good way?  Can you tell me about one time you did not think you handled asking in a good way?  Can you describe how you perform the routine enquiring?  What do you do if a woman deny being exposed the first time you ask? Do you ask again?  How does women react on the questions about violence?  Can you describe how you manage time and resources regarding asking about violence? |
| **How does your organisation work with the topic?**  How do you feel your organisation support women and the unborn child’s need for help today?  Who do you consult when you feel the need for more knowledge and support?  What is the biggest advantages at your workplace regarding this work? Disadvantages?  Do you feel that your organisation miss something?  How does your organisation take care of the need for more competence?  Do you have some suggestions that could make it easier to communicate about violence?  Thank you for your time! |

Table S2. Example of the analysis process with meaning units, codes, subthemes and theme

| **Meaning unit** | **Condensed meaning unit** | **Codes** | **Subtheme** | **Theme** |
| --- | --- | --- | --- | --- |
| Even if I still feel it is not natural (to ask), because I do. I can see the purpose if violence is uncovered. Because the statistics says many women are exposed. Of course it has a purpose. | It feels un-natural to ask, but it has a purpose because statistics tells that many are abused | Asking has a purpose | Motivation | Midwives asks about violence |
| It was scary at the beginning, to start to ask. We felt that we still needed to learn more | It was scary in the beginning to ask | Scary | Difficult to get started |  |
| I have never met anyone who have reviled that they live in a violence relationship. But the women I meet have a lot of resources and it is probably few that are exposed | No one have said yes and few are probably exposed | Few are exposed to violence among resourceful women | Knowledge and attitudes |  |
